# Lymph node involvement influences prognosis in resectable non-metastatic solid pseudopapillary tumors of the pancreas: a SEER-based population study

**DOI:** 10.1007/s13304-026-02562-5

**Published:** 2026-02-13

**Authors:** Vincenzo D’Ambra, Claudio Ricci, Laura Alberici, Carlo Ingaldi, Marco Fichera, Stefano Togni, Riccardo Casadei

**Affiliations:** 1 Division of Pancreatic Surgery, Department of Internal Medicine and Surgery (DIMEC), Alma Mater Studiorum, IRCCS, Azienda Ospedaliero-Universitaria di Bologna, University of Bologna, S. Orsola-Malpighi Hospital, Via Massarenti n.9, 40138 Bologna, Italy; 2 Department of Internal Medicine and Surgery (DIMEC), Alma Mater Studiorum, University of Bologna, S. Orsola-Malpighi Hospital, Bologna, Italy

**Keywords:** Solid pseudopapillary tumor, Pancreatic cancer, Rare pancreatic tumors

## Abstract

Solid Pseudopapillary Tumors (SPT) of the pancreas are rare neoplasms with an often indolent biological behavior. Given the rarity of this pathology, studies based on large population registries are fundamental to delineate patient profiles and survival. This study aims to analyze characteristics and survival in patients with resectable non-metastatic pancreatic SPTs utilizing a large population database. This study analyzed data from SEER database, focusing on patients with resected non-metastatic SPT of the pancreas. We excluded patients who did not undergo surgery, with inadequate lymphadenectomy, or with metastatic disease. Patients and tumors characteristics were extracted. Univariate and multivariate Cox regression analyses were performed to identify factors influencing prognosis. Kaplan-Meier curves were used to compare survival across subgroups. A contour plot and restricted cubic spline were calculated to assess the relationship between variables and hazard. The study included 293 patients, predominantly female (84.6%) with a median age of 20–40 years (44.4%). The overall 5-year OS was 96.2%. Kaplan-Meier analysis showed that patients with positive lymph-nodes had a markedly worse prognosis, as did patients with older age. Survival was also influenced by tumor location, with head or body tumors showing lower survival than tail tumors after approximately 100 months. In multivariate Cox regression, age ( *p* = 0.004), location ( *p* = 0.006), positive lymph nodes ( *p* < 0.001), and chemotherapy ( *p* < 0.001) were significant predictors. The contour plot and restricted cubic spline confirmed that predicted hazard increased with both age and the number of positive lymph nodes. This large population-based study confirms that resectable, non-metastatic SPT generally have a favorable prognosis. However, factors such as advanced age, tumor location, and, in particular, lymph node involvement are associated with a worse prognosis. These observations are crucial for risk stratification and treatment planning in this rare patient population.

## Introduction

Solid Pseudopapillary Tumors (SPTs) of the pancreas are uncommon pancreatic neoplasms, typically characterized by an indolent biological course [1]. While survival predictors for PDAC are well documented, the same predictors for SPTs remain poorly characterized in the literature [2]. Despite their generally favorable prognosis after resection, understanding the factors that influence patient outcomes is crucial, especially given the rarity of this disease [1]. Due to the limited number of cases encountered at individual institutions, large population-based studies are essential to accurately characterize patient demographics, tumor features, and survival patterns.

Previous research on SPTs has been largely based on single-center experiences or small series, which may not fully capture the diverse clinical presentations and prognostic factors [3, 4]. This study aims to address this gap by leveraging a large population database to analyze the characteristics and survival of patients with resectable, non-metastatic pancreatic SPTs. Specifically, we seek to identify demographic, pathological, and treatment-related factors that influence prognosis in this unique patient cohort. The findings from this study are expected to provide valuable insights for risk stratification and the development of tailored treatment strategies for patients with resectable SPTs of the pancreas.

## Methods

### Data source and patient selection

**Fig. 1:**
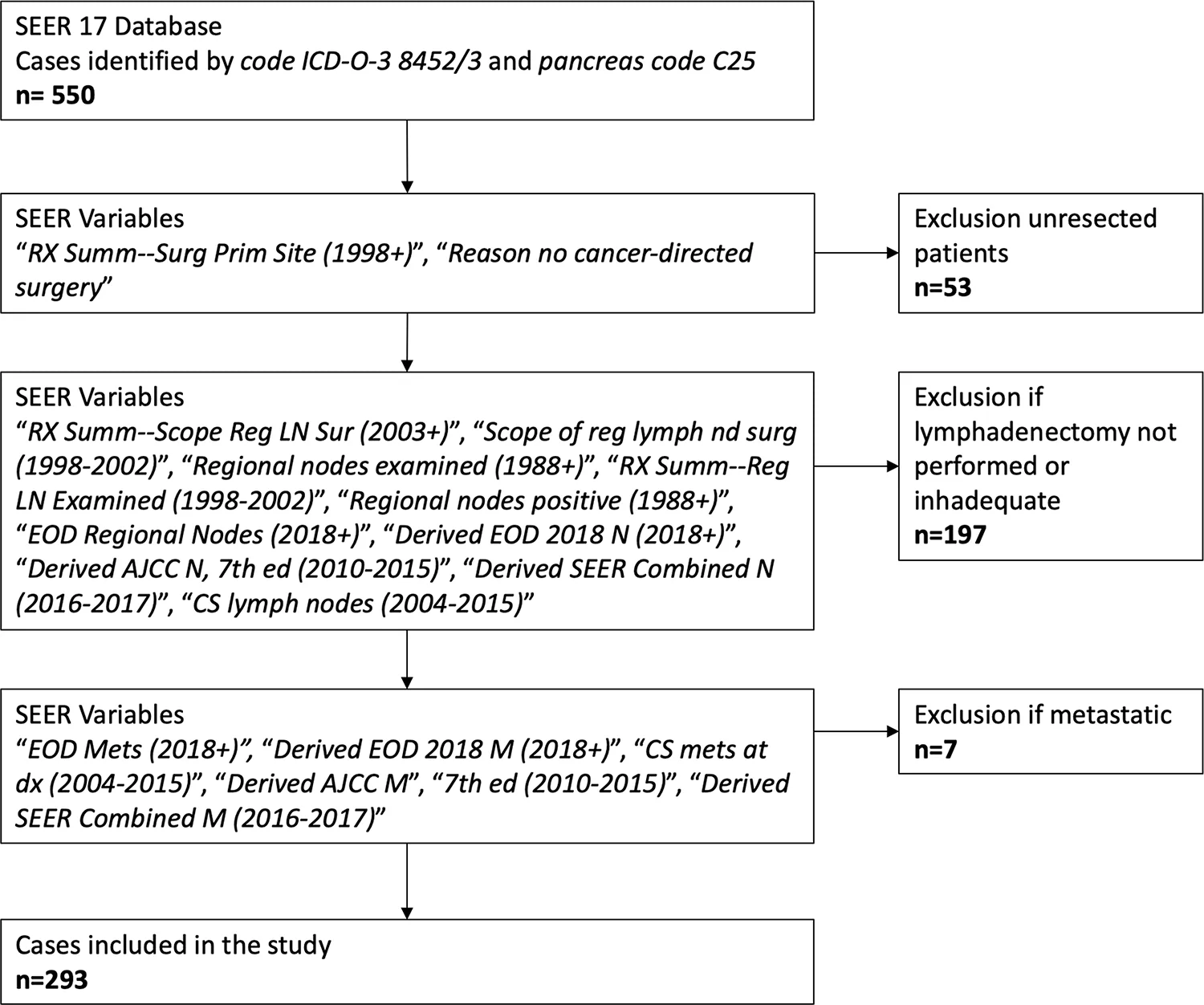
Study flow-chart of data extraction

Patients diagnosed with Solid Pseudopapillary Tumors of the pancreas from 2001 to 2025 were selected from the Surveillance, Epidemiology, and End Results (SEER) database, accessed via http://www.seer.cancer.gov, utilizing the SEER*Stat software (version 8.4.4) [5]. The SEER program collects cancer incidence and survival data from population-based registries across the United States [5]. Our analysis focused on patients diagnosed with non-metastatic Solid Pseudopapillary Tumors (SPT) of the pancreas who underwent surgical intervention. The inclusion criteria were: patients diagnosed with non-metastatic SPT of the pancreas who underwent radical surgery. On the other hand, the exclusion criteria were: surgery not performed, inadequate lymphadenectomy, histology different than SPT, metastatic disease at diagnosis. The patient selection process is described in Fig. 1. Selection of patients was based on the International Classification of Diseases for Oncology Third Edition (ICD-O-3) [6], using histology code 8452/3 (SPT), and primary anatomic site code C25 (pancreas). These selection criteria aimed to focus on a cohort of patients with resectable, non-metastatic disease, where the impact of lymph node involvement could be accurately assessed.

## Data extraction and endpoints

Key variables collected included: Hospital Area, Age at diagnosis, Sex, Tumor Location, Type of Surgery, Receipt of Radiotherapy, Receipt of Chemotherapy, Lymph-Nodes, Size of tumor, T Stage, N Stage, SEER Stage. Additionally, data on survival duration and current health status were recorded. Each patient’s pathological stage was reassessed based on SEER staging data, using the criteria provided in the AJCC 8th edition staging manual for pancreatic cancer [7, 8].

The primary endpoint of the study was to identify the prognostic factors associated with overall survival (OS), in patients with SPT. Secondary endpoints included estimation of survival probabilities among subgroups, and characterization of non‑linear relations between prognostic variables and hazard using restricted cubic splines.

### Statistical analysis

Categorical variables were reported as frequencies and percentages, continuous variables as median and range. Univariate and multivariate Cox regression analyses were performed to identify independent factors influencing prognosis. Hazard Ratios (HR) and their corresponding 95% Confidence Intervals (CI) and p-values were calculated for each variable. Kaplan-Meier survival curves were generated to visualize overall survival probabilities across different subgroups. Missing data were handled using an available-case approach. For each variable, only patients with non-missing values were included in the corresponding Cox regression analysis. To further assess the relationship between continuous variables and hazard, contour plots and restricted cubic splines were calculated. A two-tailed p-value inferior to 0.05 was considered statistically significant. All statistical analyses were performed using the software Stata SE version 18.0.

## Results

### Patient characteristics

Flow-chart of the screening process is described in Fig. 1. A total of 550 cases were identified from the SEER database by combining the SPT histology code (8452/3) with pancreatic tumor location (C25). Of these, 53 patients were excluded due to lack of surgical resection, 197 were excluded because lymphadenectomy was either not performed or inadequate, and 7 patients were excluded due to metastatic disease. Finally, a total of 293 patients were included in the study cohort. All resections included in this study were performed between 2001 and 2020. The majority of patients were female (84.6%). The median age group at diagnosis was 20–40 years, accounting for 44.4% of the cohort, followed by 40–60 years (32.4%) and 0–20 years (15.7%). Tumor location distribution showed that 43.7% were in the tail, 40.8% in the head, and 15.5% in the body of the pancreas. Pancreaticoduodenectomy was the most common surgical procedure (50.3%), followed by distal pancreatectomy (39.8%). The median number of harvested lymph nodes was 13 (range: 5–45), and the median number of positive lymph nodes was 0 (range: 0–4). The median tumor size was 50 mm (range: 4–240), with three tumors exceeding 200 mm in size; however, the IQR was 30–75 mm. The most common AJCC stage was IIA (54.9%). The majority of patients received neither chemotherapy (97.3%) nor postoperative radiotherapy (99.3%). Detailed baseline characteristics are presented in Table 1.

**Table 1:** Baseline characteristics of included patients. Excluded non-surgical patients, lymphadenectomy not performed (LN < 5), metastatic disease

Characteristics of patients (*n* = 293)	*N*(%) or median (range)
*Hospital area*	
Metropolitan > 1M	199 (68.2)
Metropolitan 250 K – 1M	68 (23.3)
Metropolitan < 250 K	8 (2.7)
Near to Metropolitan	11 (3.8)
Distant from Metropolitan	6 (2.1)
*Age*	
0–20	46 (15.7)
20–40	130 (44.4)
40–60	95 (32.4)
60–80	22 (7.5)
*Sex* Male Female	45 (15.4) 248 (84.6)
*Location*	
Head	108 (40.8)
Body	41 (15.5)
Tail	116 (43.7)
*Type of Surgery*	
Enucleoresection	4 (1.5)
Pancreaticoduodenectomy	138 (50.3)
Distal Pancreatectomy	109 (39.8)
Total Pancreatectomy	23 (8.4)
*Radiotherapy*	
Yes	2 (0.7)
No	291 (99.3)
*Chemotherapy*	
Yes	8 (2.7)
No	285 (97.3)
Harvested Lymph-Nodes	13 (5–45)
Positive Lymph-Nodes	0 (0–4)
Lymph-Node Ratio	0 (0–0.6)
Size of tumor	50 (4–240)
*T Stage*	
T1a	1 (0.3)
T1b	2 (0.7)
T1c	25 (8.7)
T2	87 (30.4)
T3	166 (58.1)
T4	5 (1.8)
*N Stage*	
N0	277 (94.5)
N1	15 (5.1)
N2	1 (0.4)
*AJCC Stage*	
Stage 1a	28 (9.8)
Stage 1b	84 (29.4)
Stage 1c	0 (0.0)
Stage 2a	157 (54.9)
Stage 2b	12 (4.2)
Stage 3	5 (1.7)

### Survival analysis

**Fig. 2:**
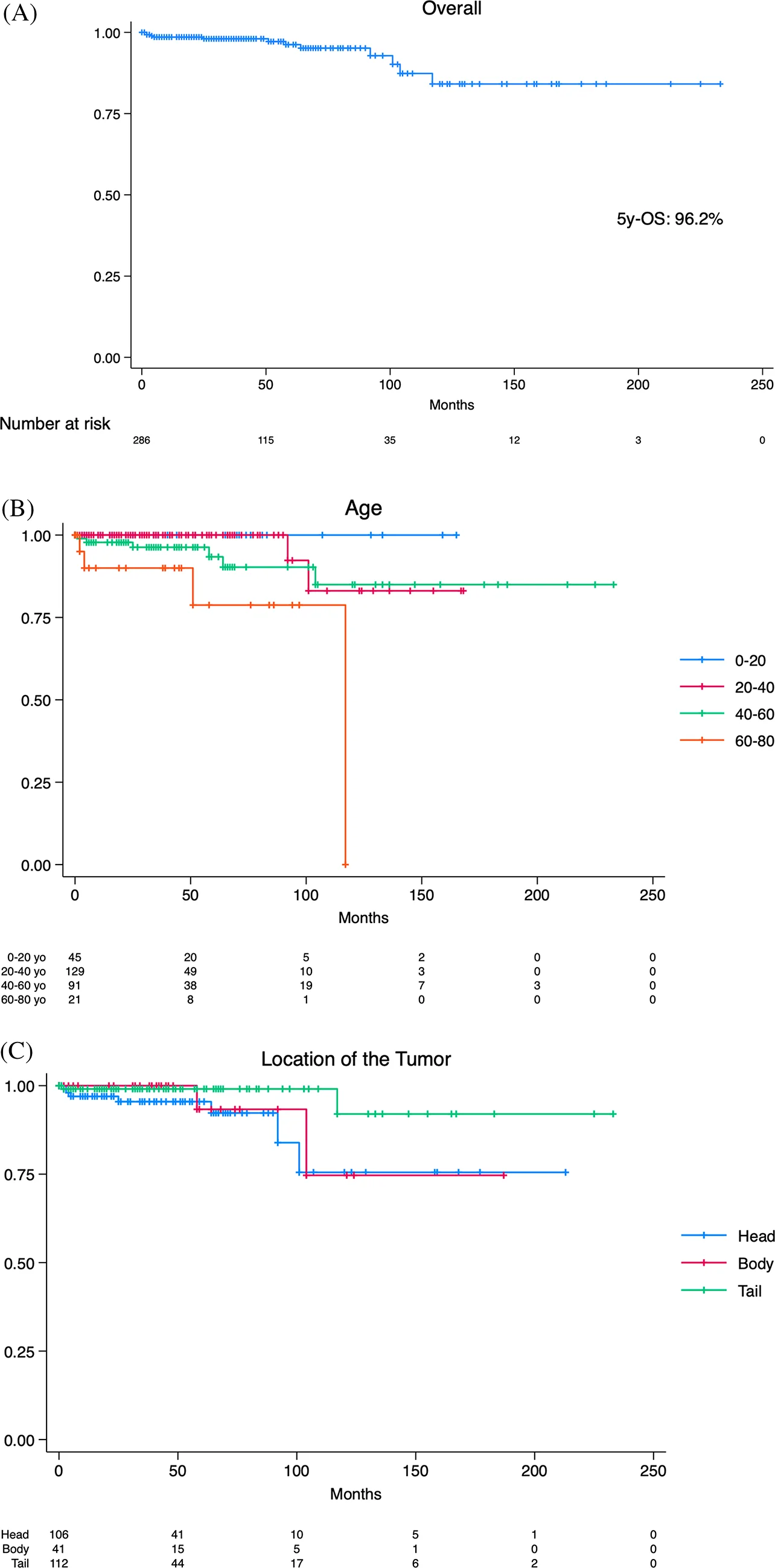

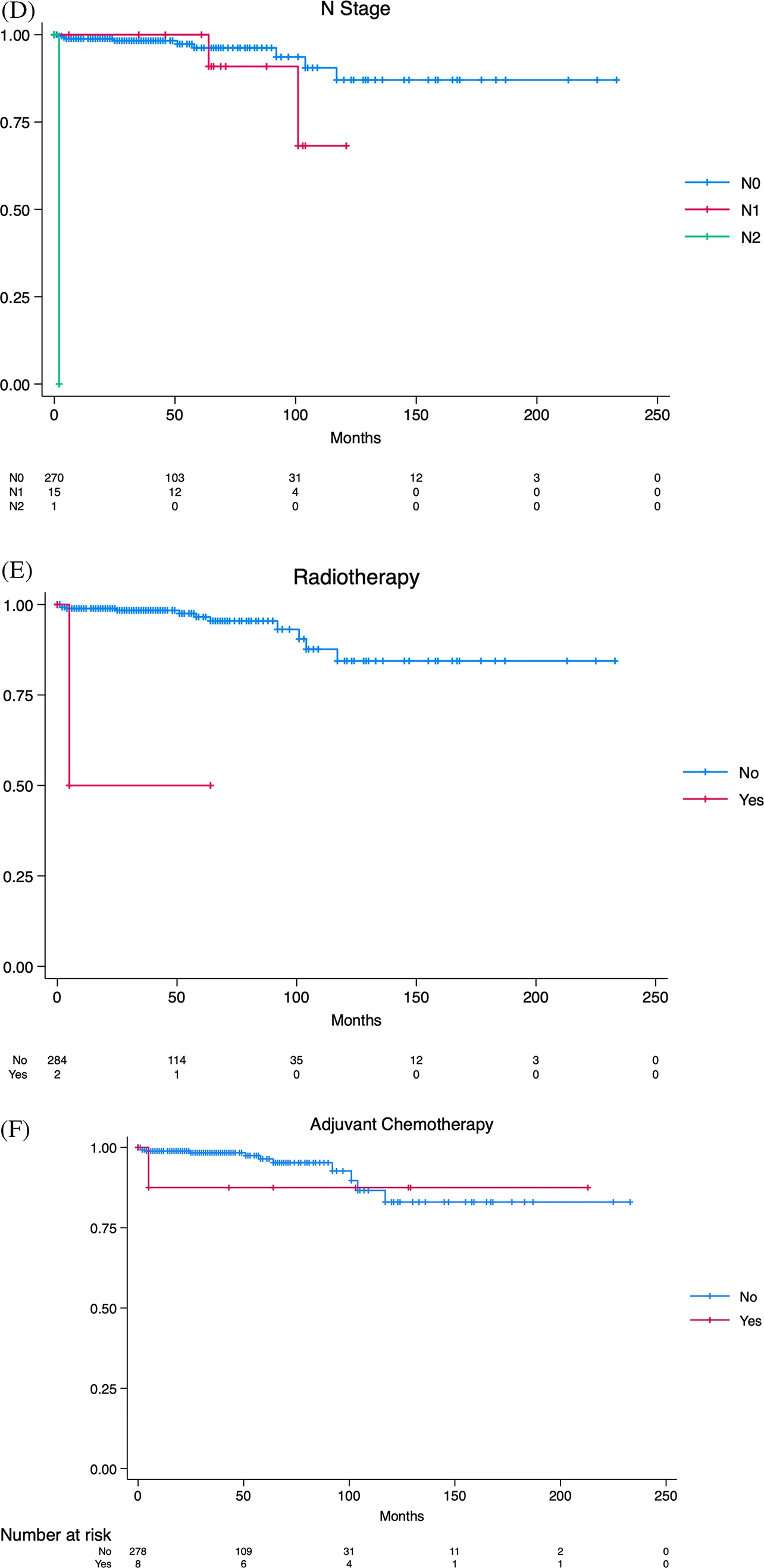
Kaplan-Meier survival curves by prognostic factors.** A** Overall Survival of the entire cohort. The 5-year Overall Survival is indicated as 96.2%;** B** Age;** C** Location of the tumor;** D** N Stage;** E** Radiotherapy administration;** F** Chemotherapy administration

The overall 5-year OS for the entire cohort was 96.2% (Fig. 2A). The results of the univariate and multivariate Cox regression analyses are summarized in Table 2. Kaplan Meyer Curves are depicted in Fig. 2. In univariate analysis, Age (HR 3.99, 95% CI 1.76–9.03, *p* = 0.001), Radiotherapy (HR 17.8, 95% CI 2.17-145.14, *p* = 0.007), Positive Lymph-Nodes (HR 2.57, 95% CI 1.46–4.51, *p* = 0.001), a high Lymph-Node Ratio ( *p* < 0.001), and N Stage (HR 5.43, 95% CI 1.72–17.17, *p* = 0.004) were significantly associated with a worse prognosis.

**Table 2:** Cox regression

Characteristics	Univariate			Multivariate		
	HR	95% CI	*p*-value	HR	95% CI	*p*-value
Hospital Area	1.46	0.90; 2.35	0.123			
Age	3.99	1.76; 9.03	**0.001**	8.29	1.95; 35.26	0.004*
Sex	0.38	0.10; 1.41	0.146			
Location	0.48	0.23; 1.00	**0.050**	0.13	0.03; 0.56	**0.006**
Type of Surgery	0.62	0.24; 1.59	0.323			
Radiotherapy	17.8	2.17; 145.14	**0.007**			
Chemotherapy	1.37	0.17; 10.91	0.763	8e-10	5e-11; 1e-8	**< 0.001**
Harvested Lymph-Nodes	0.97	0.89; 1.06	0.555			
Positive Lymph-Nodes	2.57	1.46; 4.51	**0.001**	5e30	3e29; 8e31	< 0.001*
Lymph-Node Ratio	11,147	139.5; 890k	**< 0.001**			
Size of tumor	1.00	0.99; 1.02	0.585			
T Stage	1.46	0.51; 4.16	0.480			
N Stage	5.43	1.72; 17.17	**0.004**			
AJCC Stage	1.91	0.52; 7.00	0.330			

*=selected variables after backward Cox regression

Bold indicates statistically significant p-values

In multivariate Cox regression analysis, several factors remained significant independent predictors of prognosis. In particular, higher age (HR 8.29, 95% CI 1.95–35.26, *p* = 0.004), head or body location (HR 0.13, 95% CI 0.03–0.56, *p* = 0.006), chemotherapy administration (*p* < 0.001) and positive Lymph-Nodes (*p* < 0.001) correlated with a worse prognosis.

## Age and lymph-node impact

The most important variables confirmed as independent predictors of prognosis also after a backward Cox regression analysis were Age and Positive Lymph-Nodes.

**Fig. 3:**
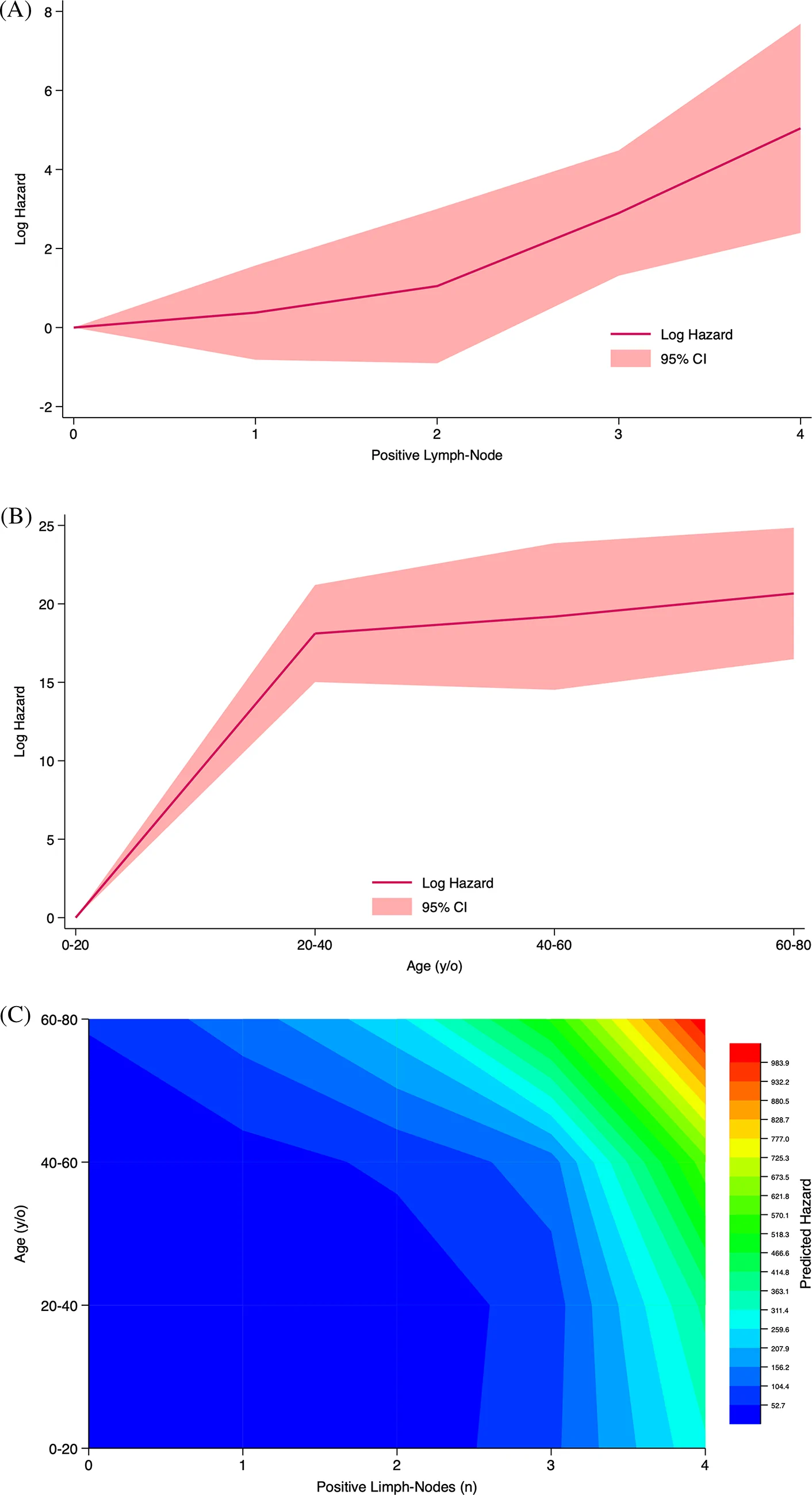
**A** Restricted Cubic Spline illustrating the absolute hazard as a function of patient’s age.** B** Restricted Cubic Spline illustrating the absolute hazard as a function of the number of positive lymph nodes.** C** Contour plot illustrating the predicted hazard as a function of both age and the number of positive lymph nodes. The color gradient indicates the level of risk, with warmer colors (red) representing higher hazard and cooler colors (blue) representing lower hazard

The contour plot and restricted cubic spline analyses further confirmed these findings. In particular, the hazard increased with age and with the number of positive lymph nodes (Fig. 3A and B). Notably Patients diagnosed at a young age have a significantly better risk profile compared to older age groups, with a clear breakpoint starting from the 20–40 age range onwards. The contour plot depicted in Fig. 3C, visually represents the simultaneous influence of these two variables, with a higher hazard in older patients and those with a greater number of positive lymph nodes.

## Discussion

This large population-based study confirms that resectable, non-metastatic Solid Pseudopapillary Tumors of the pancreas generally carry a favorable prognosis, as evidenced by an impressive overall 5-year survival rate of 96.2%. This finding is consistent with the generally indolent nature attributed to SPTs [9]. However, our comprehensive analysis, utilizing univariate and multivariate Cox regression, Kaplan-Meier survival curves, and advanced modeling techniques like restricted cubic splines and contour plots, has identified several critical factors associated with a worse prognosis.

A key finding of our study is the profound impact of lymph node involvement on survival. Both univariate and multivariate analyses consistently demonstrated that the presence of positive lymph nodes is strongly associated with poorer outcomes, although the exact magnitude of the hazard ratio in the multivariate model should be interpreted with caution due to the limited number of events. This highlights the importance of thorough lymphadenectomy and pathological examination in patients undergoing resection for SPTs [10]. Furthermore, in univariate analysis the N stage also showed a significant association with prognosis. The contour plot and restricted cubic spline reinforced this, showing a clear increase in predicted hazard with an increasing number of positive lymph nodes. This suggests that while SPTs are often considered to have low metastatic potential (only 5.5% of patients in this cohort showed positive node), nodal involvement predicts a more aggressive biological behavior and warrants careful consideration in treatment planning [10, 11].

Age at diagnosis emerged as another crucial prognostic factor. Older age groups were associated with a markedly worse prognosis in Kaplan-Meier analysis and a significantly higher hazard in both univariate and multivariate Cox regression analyses. The restricted cubic spline also depicted a clear increase in log hazard with advancing age. This observation is important for risk stratification, suggesting that older patients with SPTs may require more vigilant follow-up or consideration of more aggressive surgical management [12, 13].

Tumor location also influenced survival outcomes. Kaplan-Meier analysis showed that head or body tumors had lower survival rates compared to tail tumors after approximately 100 months. This was further supported by the multivariate Cox regression where location emerged as a significant predictor. The reasons for this differential prognosis based on location may be multifactorial, potentially related to differences in tumor biology, anatomical considerations for complete resection, lymphatic drainage patterns, or simply for a more extensive surgery that has a greater impact on patient survival [14, 15].

Interestingly, chemotherapy was identified as a significant predictor in multivariate analysis, and radiotherapy showed a significant association in univariate analysis. However, only 8 patients received chemotherapy and 2 received radiotherapy, which is an extremely small subset. The observed poorer survival among these patients likely reflects selection bias, as these treatments were offered to clinically more aggressive cases, rather than a true effect of the therapies themselves [14, 16]. Therefore, these findings should be interpreted with caution, and no definitive conclusions about the efficacy of chemotherapy or radiotherapy can be drawn.

This study has some strengths and limitations. The strengths of this study lie in its utilization of a large, population-based registry (SEER), which minimizes selection bias and provides a representative cohort of patients with SPTs. Furthermore, the use of advanced statistical methods, including restricted cubic splines and contour plots, allowed for a nuanced understanding of the continuous relationships between variables and hazard.

Despite its strengths, the limitations are mainly attributable to the retrospective nature of SEER-based analyses. In particular, information on specific surgical techniques, detailed pathological features beyond what is captured by SEER, and comprehensive data on complications or recurrence patterns are not available. Moreover, the registry-based design limits a precise correlation between tumor location and surgical procedure. In addition, SEER database does not provide details on chemotherapy type or regimen. As a result, the worse prognosis observed in the small subset of patients receiving chemotherapy likely reflects treatment selection bias (i.e., chemotherapy offered in clinically more aggressive or advanced cases), rather than a direct effect of specific agents. Another limitation is that DFS and DSS could not be analyzed due to intrinsic constraints of the SEER database. In fact, SEER does not systematically record tumor recurrence, making DFS unobtainable, and its cause-specific mortality data may be unreliable for rare tumors with very few events, such as SPTs. For these reasons, OS was selected as the primary and most robust outcome measure. Finally, some variables showed extreme hazard ratio values in the multivariable Cox regression, which is a known issue in datasets with very few events. Despite that, the direction and significance of the associations are more informative than the exact HR values.

## Conclusions

In conclusion, despite the limitations, this study reaffirms the generally favorable prognosis of resectable, non-metastatic SPTs. Nevertheless, advanced age, tumor location, and, most notably, lymph node involvement are key predictors of worse outcomes, providing guidance for risk stratification, treatment planning, and follow-up. Future prospective studies are needed to validate these findings and optimize management for higher-risk patients [17, 18].
